# Body mass index and postoperative mortality in patients undergoing coronary artery bypass graft surgery plus valve replacement: a retrospective cohort study

**DOI:** 10.7717/peerj.13601

**Published:** 2022-06-14

**Authors:** Chun Dai, Hongbo Xu, Tianshu Chu, Boyang Cao, Jianjun Ge

**Affiliations:** 1 The Lu’an Hospital Affiliated to Anhui Medical University, Lu’an, Anhui, China; 2 The Lu’an People’s Hospital, Lu’an, Anhui, China; 3 The First Affiliated Hospital of USTC, Hefei, China

**Keywords:** BMI, Coronary artery bypass graft, Valve replacement, Mortality, Cardiac disease

## Abstract

**Background:**

The relationship between body mass index (BMI) and postoperative mortality in patients who undergo coronary artery bypass graft (CABG) surgery plus valve replacement is uncertain. We aimed to investigate the association between body mass index (BMI) and postoperative mortality among patients who simultaneously underwent both CABG surgery plus valve replacement.

**Methods:**

We retrospectively analyzed 1976 patients who underwent CABG surgery at our hospital between January 2017 and April 2021, including 202 patients who underwent valve replacement surgery during the same period. We analyzed the relationship between BMI and postoperative mortality. The relationship between BMI and postoperative mortality was assessed using smooth curve fitting and a Multiple logistic regression model.

**Results:**

The results of smoothing curve fitting showed that BMI and postoperative mortality had a non-linear relationship, and the resulting curve exhibited a two-stage change and a breakpoint. Postoperative mortality is higher in patients that have a body mass index above 25 kg/m^2^ compared to patients having a body mass index between 18 and 25 kg/m^2^.

**Conclusions:**

Our study found a non-linear relationship between BMI and postoperative mortality in patients undergoing CABG plus valve replacement after adjusting for potential confounders. The causal relationship between BMI and postoperative mortality still requires further investigations.

## Introduction

The body mass index (BMI) is a standard measure used to assess obesity based on height and weight. Among Asians, a BMI between 23 and 24.9 kg/m^2^ is considered overweight, and a BMI greater than 25 kg/m^2^ is considered obesity (Anonymous, 2004). Obesity rates have been rising in both developed and developing countries over the past two decades, making it one of the most severe epidemics of the 21st century (Stevens et al., 2012; Flegal et al., 2012; Li et al., 2020). Being overweight can lead to diabetes mellitus, high blood pressure, cardiovascular disease, liver disease, pneumonia, bronchial asthma, cancer, and other related diseases. Individuals with obesity often develop heart disease and tend to undergo cardiovascular surgery more often than those with normal BMI (Habib et al., 2005). Patients who undergo cardiovascular surgery also tend to experience more perioperative adverse events and mortality than those with normal BMIs (Engel, McDonough & Smith, 2009; Gruberg et al., 2005; Hartrumpf, Kuehnel & Albes, 2017).

Due to the increasing prevalence of cardiovascular disease and improvements in cardiac surgery, more patients require coronary artery bypass graft (CABG) and simultaneous heart valve replacement (Paradis et al., 2014), which is a complicated procedure with a high mortality rate. Studies have shown that when heart valve replacement surgery and coronary artery bypass surgery are performed simultaneously, the mortality rate is twice that of valve surgery alone, and four times that of coronary artery bypass surgery alone (Edwards, Peterson & Coombs, 2001). Therefore, there is a need to distinguish high-risk groups at increased mortality risk after cardiac valve replacement plus CABG surgery; this is imperative for treatment and preoperative evaluation.

Many studies have reported that BMI is an important preoperative evaluation index that is closely related to the incidence of postoperative adverse events and mortality (Edwards, Peterson & Coombs, 2001; Akinleye et al., 2018; Pereira de Lima et al., 2020). Individuals with obesity have a high incidence of heart disease; moreover, BMI is also closely related to the mortality rate and long-term survival of patients after cardiac surgery (Ghanta et al., 2017; Aguiar et al., 2019). However, in the reports of independent factors of surgical risk of valve replacement plus CABG surgery, few analyses on BMI have been performed to date. Therefore, we conducted this retrospective cohort study.

## Material and Methods

### Study design and patient population

This retrospective cohort study assessed the relationship between BMI and postoperative death during mid-term observation in patients undergoing valve replacement plus CABG. The target independent variable was the BMI in the preoperative baseline data, and the dependent variable was postoperative death. We included all patients who underwent CABG combined with valve replacement surgery corresponding to the International Classification of Disease, 9th Revision, Clinical Modification of Operations and Procedures (ICD-9-CM) 36.1 (bypass anastomosis for heart revascularization) in combination with 35.2 (replacement of heart valve) between January 2017 and April 2021. All eligible patients from The First Affiliated Hospital of USTC, Division of Life Sciences and Medicine Electronic Information Center, were screened by a research nurse and a doctor of clinical medicine.

This study was approved by the Medical Ethics Committee of the First Affiliated Hospital of the University of Science and Technology of China and was conducted in accordance with the Declaration of Helsinki (approval number: 2021-RE-046, approval date: 24/05/2020). All participants provided written informed consent. All data were anonymized to protect the patients’ privacy. All research was conducted in accordance with relevant guidelines and regulations.

The inclusion criteria for this study were as follows: (1) Patients with a degree of stenosis greater than 70% and the need to undergo coronary artery bypass surgery to diagnose coronary heart disease and (2) availability of the patient’s complete medical record data. The exclusion criteria were as follows: (1) Patients who underwent coronary artery bypass surgery but did not have valves with moderate or higher valvular stenosis or insufficiency and did not undergo simultaneous valve replacement. (2) Patients undergoing tricuspid valve replacement. (3) Uncontrolled bleeding during the operation, death during the operation, or rapid death due to bleeding after the operation, such as in delayed left ventricular rupture after the operation.

### Definitions, endpoints, and follow-up

Baseline characteristics, including comorbidities, were obtained from Medicine Electronic Information Center, which includes ICD codes for all patients. The primary endpoint was all-cause mortality after surgery and follow-up. Vital status and date of death were telephonically acquired by a follow-up nurse until 31 February 2022. For patients who could not be reached, the household registration management department of the local police station was contacted by a doctor of clinical medicine to determine whether the patient was still alive.

The height and weight of each patient were measured upon admission. BMI was calculated as BMI = weight (kg) ÷ height^2^ (m^2^) and treated as a continuous variable. For the definition of postoperative death, we used the definition by the American Thoracic Society (ATS), that is, death during the same hospital stay after surgery or within 30 days postoperatively at any time and regardless of location (Shahian et al., 2009). In this study, we included the following data: (1) demographic data; (2) variables that may affect BMI or postoperative mortality according to existing literature; and (3) variables that may affect postoperative mortality based on our clinical experience. The following variables were used to construct a calibration model: age, sex, surgical history, pulmonary arterial hypertension, smoking status, diabetes mellitus, left ventricular ejection fraction (EF), B-type natriuretic peptide (BNP) level, extracorporeal circulation time (pump time), red blood cell (RBC) count, operation time, chronic renal disease, and a history of cerebrovascular disease. Each adjusted variable was changed to a categorical variable based on clinical guidance significance, previous literature, or the balance of data between groups.

### Treatment protocol

All patients underwent standardized surgical procedures. A brief description of the procedure is as follows: for patients undergoing mitral valve replacement plus CABG, the heart continued to function normally after establishment of cardiopulmonary bypass. Subsequently, the aorta was blocked, myocardial protective fluid was infused, and the mitral valve was replaced. For patients undergoing simple aortic valve replacement and double valve replacement, anastomosis of the distal coronary artery was first performed. The aorta was then incised, and myocardial protection solution was infused through the left and right coronary sinuses and bridging vessels, after which valve replacement was subsequently performed. After valve replacement, the proximal anastomosis of the bridging vessel was completed, and the heart resumed beating. All patients returned to the Cardiac Surgery Intensive Care Unit after the procedure for monitoring and treatment.

### Statistical analysis

Continuous variables with a normal distribution are presented as mean ± standard deviation, and categorical variables are expressed as frequencies or as percentages. The chi-squared or Fisher’s exact tests were used to assess categorical variables. Multiple logistic regression models will be used to explore the outcomes of interest and adjusted for statistically significant variables in univariate analyses. We performed nonlinear regression analysis to fit the clinical results with 18 and 25 as thresholds, and used a two-stage linear regression model and logarithmic likelihood ratio to test the 30-day mortality and threshold effect. All analyses were performed with the statistical software packages in R (R Core Team, 2020) and Empower Stats (http://www.empowerstats.com; X&Y Solutions, Inc, Boston, MA, USA). *P* < 0.05 (two-sided) was deemed statistically significant.

## Results

### Characteristics of the study population

**Figure 1:**
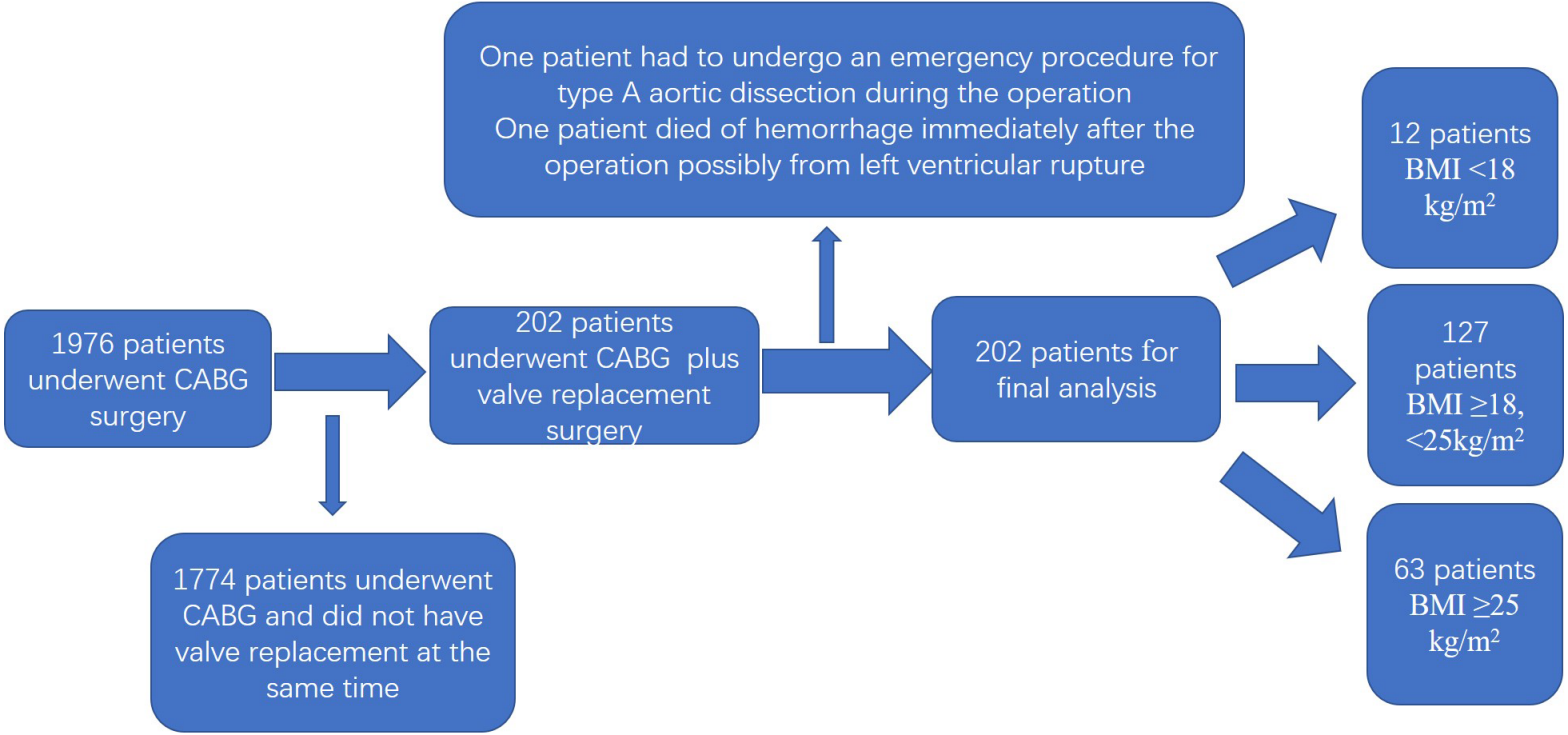
Flowchart of the study.

A total of 1,976 patients met the inclusion criteria, and 202 patients were selected for final analysis based on the exclusion criteria (Fig. 1). The baseline characteristics of the patients according to their BMI values are listed in Table 1. We grouped the participants as follows: BMI ≥25 kg/m^2^; BMI ≥18, <25 kg/m^2^ and BMI <18 kg/m^2^ based on the results of nonlinearity. The difference between the groups was statistically significant (*P* < 0.01). There were no statistical differences between the groups in terms of sex, surgical history, pulmonary arterial hypertension, smoking, diabetes mellitus, EF, BNP, extracorporeal circulation time (pump time), RBC count, operation time, chronic renal disease, or history of cerebrovascular disease.

**Table 1:** Clinical characteristics of patients by BMI.

BMI (kg/m^2^)	Total	<18	≥18, <25	≥25	*P*-value
N	202	12	127	63	
BMI (kg/m^2^)	23.4 ± 3.4	16.8 ± 0.9	22.1 ± 1.7	27.3 ± 1.7	<0.001^*^
Age (years, mean ± SD)	63.6 ± 8.6	65.9 ± 4.8	64.7 ± 8.7	60.8 ± 8.4	0.007^*^
RBC (U, mean ± SD)	4.6 ± 4.0	5.5 ± 3.8	5.2 ± 4.3	3.4 ± 2.9	0.009^*^
Pump Time (min, mean ± SD)	156 ± 48	154 ± 47	156 ± 52	157 ± 40	0.981
Cross. clamp. time (min, mean ± SD)	67.7 ± 30.1	71.7 ± 23.2	69.2 ± 30.5	63.9 ± 30.6	0.471
BNP (pg/ml, mean ± SD)	1996 ± 2821	3477 ± 3004	2157 ± 3202	1358 ± 1704	0.122
BUN (mmol/L, mean ± SD)	15.6 ± 60.2	9.9 ± 5.7	19.7 ± 76.0	8.6 ± 3.6	0.486
PH (mmHg, mean ± SD)	40.0 ± 17.2	37.3 ± 9.9	41.1 ± 17.9	38.2 ± 16.9	0.466
EF (%, mean ± SD)	61.0 ± 9.9	58.0 ± 11.4	61.7 ± 9.8	60.3 ± 9.7	0.356
Operation time (min, mean ± SD)	6.1 ± 2.7	8.1 ± 7.9	5.8 ± 1.7	6.4 ± 2.5	0.012^*^
Surgery history					0.737
No, *n* (%)	160 (79.6%)	10 (83.3%)	101 (79.5%)	49 (79.0%)	
Valve, *n* (%)	3 (1.5%)	0 (0.0%)	1 (0.8%)	2 (3.2%)	
Other, *n* (%)	38 (18.9%)	2 (16.7%)	25 (19.7%)	11 (17.7%)	
History of cerebrovascular disease					0.577
No, *n* (%)	166 (82.2%)	11 (91.7%)	105 (82.7%)	50 (79.4%)	
Yes, *n* (%)	36 (17.8%)	1 (8.3%)	22 (17.3%)	13 (20.6%)	
Chronic renal failure					0.788
No, *n* (%)	187 (92.6%)	12 (100.0%)	116 (91.3%)	59 (93.7%)	
Yes, *n* (%)	14 (6.9%)	0 (0.0%)	10 (7.9%)	4 (6.3%)	
Diabetes mellitus.					0.144
No, *n* (%)	175 (86.6%)	12 (100.0%)	112 (88.2%)	51 (81.0%)	
Yes, *n* (%)	27 (13.4%)	0 (0.0%)	15 (11.8%)	12 (19.0%)	
Smoking					0.802
No, *n* (%)	168 (83.2%)	10 (83.3%)	104 (81.9%)	54 (85.7%)	
Yes, *n* (%)	34 (16.8%)	2 (16.7%)	23 (18.1%)	9 (14.3%)	
Sex.					0.068
Female, *n* (%)	74 (36.6%)	6 (50.0%)	52 (40.9%)	16 (25.4%)	
Male, *n* (%)	128 (63.4%)	6 (50.0%)	75 (59.1%)	47 (74.6%)	
Mortality within 30 days					0.001^*^
No, *n* (%)	185 (91.6%)	9 (75.0%)	123 (96.9%)	53 (84.1%)	
Yes, *n* (%)	17 (8.4%)	3 (25.0%)	4 (3.1%)	10 (15.9%)	
Age (years)					0.025^*^
<60	57 (28.2%)	1 (8.3%)	31 (24.4%)	25 (39.7%)	
≥60	145 (71.8%)	11 (91.7%)	96 (75.6%)	38 (60.3%)	
EF (%)					0.173
<55	49 (24.4%)	5 (41.7%)	26 (20.6%)	18 (28.6%)	
≥55	152 (75.6%)	7 (58.3%)	100 (79.4%)	45 (71.4%)	

**Notes.**

Abbreviations BMI body mass index PH pulmonary arterial hypertension RBC red blood cell EF ejection fraction BNP B-type natriuretic peptide (* *p* < 0.05)

### Univariate analysis

The results of univariate analyses are listed in Table 2. The results of univariate analysis showed that BMI was significantly associated with postoperative mortality (*p* < 0.05). Without adjusting for other variables, per univariate analysis, male patients, those aged >60 years, smokers, those with diabetes mellitus, those with an EF of ≥55%, and those with longer pump time tended to have a higher risk of postoperative mortality but did not reach significance level (Table 2).

**Table 2:** Univariate analysis of patient mortality.

Mortality	Statistics	OR (95% CI)	*p*-value
Surgery history			
No, *n* (%)	160 (79.6%)	1.0	
Valve, *n* (%)	3 (1.5%)	0.0 (0.0, Inf)	0.992
Other, *n* (%)	38 (18.9%)	0.5 (0.1, 2.5)	0.423
History of cerebrovascular disease			
No, *n* (%)	166 (82.2%)	1.0	
Yes, *n* (%)	36 (17.8%)	2.8 (1.0, 8.2)	0.057
Chronic renal failure			
No, *n* (%)	187 (92.6%)	1.0	
Yes, *n* (%)	14 (6.9%)	0.8 (0.1, 6.7)	0.855
Diabetes mellitus			
No, *n* (%)	175 (86.6%)	1.0	
Yes, *n* (%)	27 (13.4%)	3.1 (1.0, 9.6)	0.051
Smoking			
No, *n* (%)	168 (83.2%)	1.0	
Yes, *n* (%)	34 (16.8%)	0.3 (0.0, 2.2)	0.235
Sex.			
Female, *n* (%)	74 (36.6%)	1.0	
Male, *n* (%)	128 (63.4%)	0.6 (0.2, 1.7)	0.355
RBC (U, mean ± SD)	4.6 ± 4.0	1.0 (0.9, 1.2)	0.410
Pump. time (min, mean ± SD)	156.3 ± 48.1	1.0 (1.0, 1.0)	0.057
Cross. clamp. time (min, mean ± SD)	67.7 ± 30.1	1.0 (1.0, 1.0)	0.009 ^∗^
BNP (pg/ml, mean ± SD)	1996 ± 2821	1.0 (1.0, 1.0)	0.598
BUN (mmol/L, mean ± SD)	15.6 ± 60.2	1.0 (1.0, 1.0)	0.742
PH (mmHg, mean ± SD)	40.0 ± 17.2	1.0 (1.0, 1.0)	0.994
BMI (kg/m^2^)	23.4 ± 3.4	1.2 (1.0, 1.4)	0.028 ^∗^
<18 (%)	12 (5.9%)	1.0	
≥18, <25 (%)	127 (62.9%)	0.1 (0.0, 0.5)	0.005 ^∗^
≥25 (%)	63 (31.2%)	0.6 (0.1, 2.5)	0.448
Age (years, mean ± SD)	63.6 ± 8.6	1.0 (0.9, 1.1)	0.866
<60 (%)	57 (28.2%)	1.0	
≥60 (%)	145 (71.8%)	0.9 (0.3, 2.8)	0.909
EF (%, mean ± SD)	61.0 ± 9.9	1.0 (0.9, 1.0)	0.207
<55 (%)	49 (24.4%)	1.0	
≥55 (%)	152 (75.6%)	0.4 (0.2, 1.2)	0.100

**Notes.**

Abbreviations OR odds ratio CI confidence interval PH pulmonary arterial hypertension RBC red blood cell EF ejection fraction (*: *p* < 0.05)

### Results of crude and adjusted models

To investigate the relationship between BMI and mortality, a multivariate analysis was conducted. We built different models by adjusting for different confounding factors. Model I adjusted for the baseline information of patients, including age, sex, and smoking, and Model II added all confounding factors, including age, sex, smoking, diabetes mellitus, extracorporeal circulation time (pump time), history of cerebrovascular disease, chronic renal disease, surgical history, operation time, pulmonary arterial hypertension, EF, and RBC count. The results are shown in Table 3. In the unadjusted model (crude model), patients with BMI ≥25 kg/m^2^ had a 4.8-times higher risk of death than patients with BMI ≥18, <25 kg/m^2^. In the fully adjusted model, patients with BMI ≥25 kg/m^2^ had an 11.5-times higher risk of death than patients with BMI <25 kg/m^2^.

**Table 3:** Relationship between BMI and mortality in different models.

Exposure	Crude model	Minimally adjusted model	Fully adjusted model
	OR (95% CI), *p*-value	OR (95% CI), *p*-value	OR (95% CI), *p*-value
BMI (kg/m^2^, mean ± SD)	1.2 (1.0, 1.4) 0.028	1.2 (1.0, 1.4) 0.023	1.2 (1.0, 1.5) 0.048
BMI (kg/m^2^)			
[18, 25)	Reference	Reference	Reference
<18	10.2 (2.0, 53.0) 0.005	9.9 (1.9, 52.6) 0.007	24.3 (3.0, 197.9) 0.003
≥25	5.8 (1.7, 19.3) 0.004	6.6 (1.9, 23.7) 0.004	12.5 (2.3, 68.4) 0.004

**Notes.**

Crude model: We did not adjust for any covariates.

Minimally adjusted model: We adjusted for age, sex, and smoking.

Fully adjusted model: We adjusted for Surgery history; History of cerebrovascular disease; Chronic renal failure; Diabetes.; Smoking; Sex.; Age; RBC.U Pump time; Cross. clamp. Time; Operation time; PH; EF.

### Results of nonlinearity

**Figure 2:**
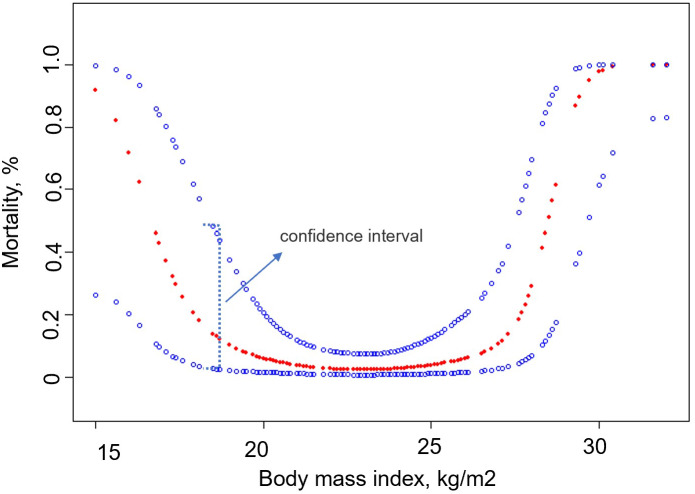
Association between body mass index and 30-day mortality.

We analyzed the relationship between BMI and 30-day mortality after smooth curve fitting (Fig. 2). The results showed that before the inflection point, the 30-day mortality rate of patients decreased with an increase in BMI, and after the inflection point was reached, the 30-day mortality rate increased with an increase in BMI. Using threshold effect analysis, the inflection points of the 30-day mortality after surgery were determined to be 18 and 25 kg/m^2^ (Table 4). The log-likelihood ratio test value was less than 0.001.

**Table 4:** Threshold effect analysis.

Outcome:	Mortality within 30 days	*p*-value
	OR (95% CI)	
Linear effect	1.2 (1.0, 1.5)	0.048
Inflection point of BMI (kg/m^2^)	18, 25	
<18 kg/m^2^	0.2 (0.0, 1.1)	0.062
[18, 25) kg/m^2^	0.7 (0.4, 1.3)	0.277
≥25 kg/m^2^	4.2 (1.6, 11.5)	0.005
Logarithmic likelihood ratio test	<0.001	

**Notes.**

Adjusted for the following variables: Surgery history; History of cerebrovascular disease; Chronic renal failure; Sex; Age; PH; EF; Smoking; Pump time; Cross. clamp. Time.

Abbreviations OR odds ratio CI confidence interval BMI body mass index; pulmonary arterial hypertension RBC red blood cell EF ejection fraction

## Discussion

In this study, we observed a non-linear relationship between BMI and the 30-day postoperative mortality of patients who underwent simultaneous valve replacement plus bypass. Patients with BMI in the normal range had lower postoperative mortality compared to patients with BMI outside of the normal range. However, when the patient’s BMI was ≥25 kg/m^2^, the postoperative mortality rate was positively correlated with BMI. In addition, after adjusting for confounding factors, we found that the postoperative mortality of patients with BMI ≥25 kg/m^2^ was 11.5 times that of patients with BMI between 18 and 25 kg/m^2^.

Studies have shown that patients who undergo CABG plus valve replacement surgery simultaneously have higher postoperative mortality (Davarpas, Hosseinsabet & Jalali, 2015). The combined procedure is more complicated than performing CABG alone. The procedure usually requires prolonged aortic cross-clamping and is accompanied by serious complications (Shaikh et al., 2011). Several studies have demonstrated that there is a concerted effort to reduce the incidence of adverse events after CABG combined with valve replacement surgery (Ricci et al., 2009; Hausenloy et al., 2015). Despite the latest advances in surgical techniques, anesthesia, and postoperative care, the postoperative mortality rate of simultaneous CABG surgery plus valve replacement is still higher than that of CABG surgery alone (Ghanta et al., 2017). Therefore, it is crucial to analyze the independent risk factors of mortality after CABG surgery combined with valve replacement. The incidence of obesity is rising rapidly worldwide (Malhotra et al., 2020; Rosenstock et al., 2018; Huseini et al., 2014). Studies have shown that patients with obesity not only have a high incidence of heart disease, but also often experience adverse events and mortality following cardiac surgery (Nicolini et al., 2014). Studies have shown that the postoperative mortality of patients with obesity who undergo CABG surgery is significantly higher than that of patients of normal weight. Some scholars have also proposed the existence of the obesity paradox (Gruberg et al., 2005). Their research has shown that the incidence of adverse events and mortality after cardiac surgery in patients with obesity is lower than that of normal patients. Many studies have reported that the outcomes of CABG surgery or valve replacement surgery alone are closely related to BMI (Burns et al., 2021; Mariscalco et al., 2017). However, few studies on whether the postoperative mortality of simultaneous CABG plus valve replacement surgery is related to BMI have been conducted to date.

Our study population included patients who required both CABG surgery plus valve replacement. The postoperative mortality rate of this operation is relatively high, and few studies have reported BMI as an independent risk factor for this operation to date, even though clinicians mostly believe that patients with high BMIs experience a higher incidence of adverse events and postoperative mortality. However, the specific risk value of BMI has not been determined. Our research results showed that the 30-day postoperative mortality rate was significantly increased among patients with BMI ≥25 and BMI <18 kg/m^2^, which provides a reference for clinical evaluation. When a patient’s BMI is ≥25 kg/m^2^, we must bear in mind that interventional valve replacement with a beating heart may be a better choice, and the BMI between 18 to 25 kg/m^2^, if conditions permit, can effectively reduce the postoperative mortality.

Although the results of this study are compelling, there are still some limitations that need to be addressed. First, this was a retrospective study and some patients’ records were missing, which may have resulted in patient selection bias. Second, the study was conducted in a single medical center with a relatively small sample, which may not represent the general characteristics of the population. The third limitation is that our model has not been validated by other groups. In the future, we plan to conduct a validation study in other centers and explore the relationship between BMI and perioperative or postoperative mortality.

## Conclusions

In conclusion, we described the non-linear relationship between BMI and 30-day mortality in patients who underwent both CABG surgery plus valve replacement after adjusting for potential confounding factors. When the BMI is ≥25 kg/m^2^, the postoperative mortality is positively correlated to the BMI and BMI is <18 kg/m^2^, postoperative mortality was negatively correlated with BMI. However, these findings do not necessarily imply a causal relationship. To show a causal connection further studies are required.

##  Supplemental Information

10.7717/peerj.13601/supp-1 Supplemental Information 1 Supplementary materials

10.7717/peerj.13601/supp-2 Supplemental Information 2 Preliminary statistics, excluding the packing standard

10.7717/peerj.13601/supp-3 Supplemental Information 3 Raw data, detailed statistics All the collected patient data

10.7717/peerj.13601/supp-4 Supplemental Information 4 Calculation data

## Additional Information and Declarations

### Data Availability

The following information was supplied regarding data availability: The raw data are available in the Supplemental Files.
