# Supplementary material for: Body mass index and postoperative mortality in patients undergoing coronary artery bypass graft surgery plus valve replacement: a retrospective cohort study
Source: PeerJ. 2022 Jun 14;10:e13601. doi: 10.7717/peerj.13601 (PMC9205315; doi:10.7717/peerj.13601)
Supplement: Supplemental Information 1 [file peerj-10-13601-s001.docx]

**S1: stratification analysis**

| Mortality | N | OR (95% CI) | p-Value |
| --- | --- | --- | --- |
| BMI(kg/m^2^) |  |  |  |
| <18 | 12 | 0.5 (0.1, 2.5) | 0.437 |
| >=18, <25 | 127 | 0.8 (0.4, 1.4) | 0.351 |
| >=25 | 63 | 2.6 (1.5, 4.6) | 0.001 |
| Surgery history |  |  |  |
| No | 160 | 1.2 (1.0, 1.4) | 0.030 |
| Valve | 3 |  |  |
| Other | 38 | 1.1 (0.7, 1.8) | 0.559 |
| History of cerebrovascular disease |  |  |  |
| No | 166 | 1.2 (1.0, 1.4) | 0.080 |
| Yes | 36 | 1.2 (0.9, 1.5) | 0.322 |
| Chronic renal failure |  |  |  |
| No | 187 | 1.2 (1.0, 1.4) | 0.054 |
| Yes | 14 | 1.6 (0.6, 4.3) | 0.331 |
| Diabetes |  |  |  |
| No | 175 | 1.1 (0.9, 1.3) | 0.305 |
| Yes | 27 | 1.4 (1.0, 2.0) | 0.058 |
| Smoking |  |  |  |
| No | 168 | 1.2 (1.0, 1.4) | 0.042 |
| Yes | 34 | 1.4 (0.7, 2.9) | 0.352 |
| Sex. |  |  |  |
| Female | 74 | 1.1 (0.9, 1.4) | 0.205 |
| Male | 128 | 1.3 (1.0, 1.6) | 0.046 |
| Age (years,) |  |  |  |
| 18 - 60 | 60 | 1.4 (1.0, 2.0) | 0.048 |
| 61 - 67 | 72 | 1.3 (1.0, 1.7) | 0.070 |
| 68 - 82 | 70 | 1.0 (0.8, 1.3) | 0.951 |
| RBC(U) |  |  |  |
| 0 - 2.5 | 58 | 40.7 (0.3, 6047.8) | 0.146 |
| 3 - 4.5 | 74 | 1.1 (0.9, 1.3) | 0.506 |
| 5 - 22 | 70 | 1.1 (0.9, 1.4) | 0.266 |
| Pump. time (min) |  |  |  |
| 2.3 - 135 | 65 | 0.9 (0.6, 1.4) | 0.709 |
| 136 - 174 | 67 | 1.3 (1.0, 1.6) | 0.045 |
| 175 - 360 | 66 | 1.1 (0.9, 1.5) | 0.289 |
| Cross. clamp. time (min) |  |  |  |
| 27 - 50 | 62 | 0.9 (0.7, 1.4) | 0.737 |
| 51 - 73 | 68 | 1.6 (1.1, 2.3) | 0.014 |
| 74 - 210 | 68 | 1.1 (0.9, 1.4) | 0.294 |
| BNP |  |  |  |
| 18.19 - 647 | 36 | 2.1 (1.0, 4.4) | 0.056 |
| 651 - 1600 | 36 | 0.9 (0.6, 1.3) | 0.462 |
| 1608 - 21893 | 36 | 0.9 (0.6, 1.3) | 0.500 |
| BUN |  |  |  |
| 3 - 5.65 | 35 | 1.1 (0.8, 1.5) | 0.657 |
| 6 - 9.49 | 88 | 1.4 (1.0, 1.7) | 0.021 |
| 10 - 709 | 66 | 1.0 (0.8, 1.3) | 0.828 |
| PH (mmHg) |  |  |  |
| 1 - 31 | 62 | 1.3 (0.9, 1.8) | 0.173 |
| 32 - 41 | 69 | 1.1 (0.9, 1.4) | 0.404 |
| 42 - 110 | 70 | 1.2 (1.0, 1.6) | 0.083 |
| EF (%) |  |  |  |
| 31 - 58 | 64 | 1.4 (1.1, 1.7) | 0.008 |
| 59 - 65 | 59 | 0.5 (0.2, 1.0) | 0.061 |
| 66 - 78 | 78 | 1.1 (0.9, 1.5) | 0.373 |
| Operation time(min) |  |  |  |
| 2.3 - 5.1 | 63 | 1.1 (0.9, 1.4) | 0.275 |
| 5.2 - 6.7 | 71 | 1.3 (0.9, 1.8) | 0.206 |
| 6.75 - 33 | 68 | 1.3 (1.0, 1.7) | 0.104 |

**S2: colinear screening**

|  | Step 1 |
| --- | --- |
| BMI(kg/m^2^) | **1.4** |
| Surgery history | **1.3** |
| History of cerebrovascular disease | **1.3** |
| Chronic renal failure | **1.5** |
| Diabetes | **1.2** |
| Smoking | **1.3** |
| Sex. | **1.2** |
| Age(years) | **1.4** |
| RBC.U | **1.5** |
| Pump. time(min) | **1.3** |
| Cross. clamp. time(min) | **1.2** |
| BNP | **1.6** |
| BUN | **1.2** |
| PH(mmHg) | **1.2** |
| EF（%） | **1.2** |
| Operation time(min) | **1.3** |

**S3: Covariate detection and screening**

| Covariates | beta | Se. | exp(beta) | 95%CI Low | 95%CI Upp | P.value |
| --- | --- | --- | --- | --- | --- | --- |
| Surgery history | -14.2974 | 1385.3778 | 0.0000 | 0.0000 | Inf | 0.9918 |
|  | -0.6217 | 0.7755 | 0.5370 | 0.1175 | 2.4552 | 0.4227 |
| History of cerebrovascular disease | 1.0361 | 0.5453 | 2.8182 | 0.9678 | 8.2062 | 0.0574 |
| Chronic renal failure | -0.1959 | 1.0702 | 0.8221 | 0.1009 | 6.6970 | 0.8548 |
|  | -13.1970 | 1455.3976 | 0.0000 | 0.0000 | Inf | 0.9928 |
| Diabetes | 1.1272 | 0.5787 | 3.0871 | 0.9930 | 9.5979 | 0.0514 |
| Smoking | -1.2452 | 1.0485 | 0.2879 | 0.0369 | 2.2476 | 0.2350 |
| Sex. | -0.4717 | 0.5096 | 0.6239 | 0.2298 | 1.6940 | 0.3546 |
| Age(years) | -0.0049 | 0.0292 | 0.9951 | 0.9398 | 1.0537 | 0.8663 |
| RBC.U | 0.0476 | 0.0578 | 1.0487 | 0.9364 | 1.1746 | 0.4105 |
| Pump. time(min) | 0.0094 | 0.0049 | 1.0094 | 0.9997 | 1.0192 | 0.0571 |
| Cross. clamp. time(min) | 0.0177 | 0.0068 | 1.0179 | 1.0045 | 1.0314 | 0.0087 |
| BNP | -0.0001 | 0.0002 | 0.9999 | 0.9995 | 1.0003 | 0.5975 |
| BUN | -0.0044 | 0.0134 | 0.9956 | 0.9698 | 1.0221 | 0.7424 |
| PH(mmHg) | 0.0001 | 0.0148 | 1.0001 | 0.9716 | 1.0295 | 0.9940 |
| EF（%） | -0.0302 | 0.0239 | 0.9703 | 0.9258 | 1.0168 | 0.2069 |
| Operation time(min) | 0.0440 | 0.0721 | 1.0450 | 0.9072 | 1.2036 | 0.5419 |

Note: Represents a change of more than 10% from the initial regression coefficient.

**S4:** **Covariates after screening**

| Y | X | Adjust I | Adjust II |
| --- | --- | --- | --- |
| Mortality within 30 days | **BMI** | **Surgery history**  **Cerebrovascular disease**  **Chronic renal failure**  **Diabetes**  **Smoking**  **Sex**  **Age**  **RBC.U**  **Pump. time**  **Cross. clamp. time**  **BNP**  **BUN**  **PH**  **EF**  **Operation time** | **Surgery history**  **Cerebrovascular disease**  **Chronic renal failure**  **Diabetes**  **Smoking**  **Sex**  **Age**  **RBC.U**  **Pump. time**  **Cross. clamp. time**  **BNP**  **BUN**  **PH**  **EF**  **Operation time** |

Note:

1. Criterion 1: Introducing covariates in the basic model or excluding covariates from the complete model has an impact on the regression coefficient of X> 10%

2. Criterion 2: Criterion 1 or the regression coefficient P value of covariates on Y <0.1
