# Supplementary material for: Body mass index and postoperative mortality in patients undergoing coronary artery bypass graft surgery plus valve replacement: a retrospective cohort study
Source: PeerJ. 2022 Jun 14;10:e13601. doi: 10.7717/peerj.13601 (PMC9205315; doi:10.7717/peerj.13601)
Supplement: Supplemental Information 4 [file peerj-10-13601-s004.zip › 3/1_1_tbl/1_1_tbl.htm]

## Æ½»¬ÇúÏßÄâºÏ

|  |
| --- |
| X1.MORT.OPERATIVE.MORTALITY.0.NONE.1YES vs. BODY.MASS.INDEX |

Generalize additive models
Outcome: X1.MORT.OPERATIVE.MORTALITY.0.NONE.1YES
Exposure: BODY.MASS.INDEX
Linear terms effect

|  |  |  |  |  |  |  |  |
| --- | --- | --- | --- | --- | --- | --- | --- |
|  | Estimate | Std. Error | z value | Pr(>|z|) | exp(est) | 95%CI low | 95%CI upp |
| (Intercept) | -10.2639 | 5.0358 | -2.0382 | 0.0415 | 0 | 0 | 0.6746 |
| factor(PRIOR.SURGERY.0NO.1CABG.2VALVE.3OTHER)2 | -33.715 | 38745320.6954 | 0 | 1 | 0 | 0 | Inf |
| factor(PRIOR.SURGERY.0NO.1CABG.2VALVE.3OTHER)3 | -0.5719 | 1.0327 | -0.5537 | 0.5798 | 0.5645 | 0.0746 | 4.2727 |
| CEREBROVASCULAR.DISEASE.0NO.1YES | 0.9752 | 0.8517 | 1.1451 | 0.2522 | 2.6518 | 0.4995 | 14.0776 |
| factor(CHRONIC.RENAL.FAILURE.0NO.1YES)1 | -2.0863 | 1.6345 | -1.2764 | 0.2018 | 0.1241 | 0.005 | 3.0568 |
| factor(CHRONIC.RENAL.FAILURE.0NO.1YES)2 | -31.7132 | 67108864 | 0 | 1 | 0 | 0 | Inf |
| DIABETES.0NO.1YES | 2.8948 | 1.1667 | 2.4812 | 0.0131 | 18.0807 | 1.8369 | 177.973 |
| SMOKING.YES.0NO.1YES | -0.9385 | 1.3209 | -0.7106 | 0.4774 | 0.3912 | 0.0294 | 5.2088 |
| SEX.0.FEMALE.1.MALE | -1.1849 | 0.9096 | -1.3027 | 0.1927 | 0.3058 | 0.0514 | 1.8181 |
| AGE | 0.1165 | 0.066 | 1.7658 | 0.0774 | 1.1236 | 0.9873 | 1.2788 |
| RBC.U | 0.053 | 0.0986 | 0.5377 | 0.5908 | 1.0545 | 0.8691 | 1.2793 |
| PUMP.TIME | 0.0143 | 0.009 | 1.6011 | 0.1093 | 1.0144 | 0.9968 | 1.0324 |
| CROSS.CLAMP.TIME | 0.0247 | 0.0124 | 1.9947 | 0.0461 | 1.025 | 1.0004 | 1.0501 |
| PH | -0.0113 | 0.0255 | -0.4448 | 0.6565 | 0.9887 | 0.9406 | 1.0393 |
| EF | -0.061 | 0.0416 | -1.4649 | 0.1429 | 0.9408 | 0.8671 | 1.0208 |
| OPERATION.TIME | -0.1224 | 0.151 | -0.8104 | 0.4177 | 0.8848 | 0.6582 | 1.1895 |

Chi-square tests for linear terms

|  |  |  |  |
| --- | --- | --- | --- |
|  | df | Chi.sq | p-value |
| factor(PRIOR.SURGERY.0NO.1CABG.2VALVE.3OTHER) | 1 | 0 | 1 |
| CEREBROVASCULAR.DISEASE.0NO.1YES | 1 | 1.3112 | 0.2522 |
| factor(CHRONIC.RENAL.FAILURE.0NO.1YES) | 1 | 0 | 1 |
| DIABETES.0NO.1YES | 1 | 6.1562 | 0.0131 |
| SMOKING.YES.0NO.1YES | 1 | 0.5049 | 0.4774 |
| SEX.0.FEMALE.1.MALE | 1 | 1.6971 | 0.1927 |
| AGE | 1 | 3.1182 | 0.0774 |
| RBC.U | 1 | 0.2892 | 0.5908 |
| PUMP.TIME | 1 | 2.5636 | 0.1093 |
| CROSS.CLAMP.TIME | 1 | 3.9787 | 0.0461 |
| PH | 1 | 0.1978 | 0.6565 |
| EF | 1 | 2.146 | 0.1429 |
| OPERATION.TIME | 1 | 0.6567 | 0.4177 |

Approximate significance of smooth terms

|  |  |  |  |  |
| --- | --- | --- | --- | --- |
|  | edf | Ref.df | Chi.sq | p-value |
| s(BODY.MASS.INDEX) | 4.1351 | 5.1096 | 16.2286 | 0.0073 |

Model statistics

|  |  |
| --- | --- |
| N: | 196 |
| Adj. r-square: | 0.4788 |
| Deviance explained: | 0.5394 |
| UBRE score (sp.criterion): | -0.5228 |
| Scale estimate: | 1 |
| family: | binomial |
| link function: | logit |

Created by EmpowerStats (www.empowerstats.com) and R on 2022-03-21
