# Supplementary material for: Body mass index and postoperative mortality in patients undergoing coronary artery bypass graft surgery plus valve replacement: a retrospective cohort study
Source: PeerJ. 2022 Jun 14;10:e13601. doi: 10.7717/peerj.13601 (PMC9205315; doi:10.7717/peerj.13601)
Supplement: Supplemental Information 4 [file peerj-10-13601-s004.zip › 3/1_2_tbl/1_2_tbl.htm]

## ¶à¸ö»Ø¹é·½³Ì

|  |  |  |  |
| --- | --- | --- | --- |
| Exposure | Non-adjusted | Adjust I | Adjust II |
| BODY.MASS.INDEX | 1.2 (1.0, 1.4) 0.028 | 1.2 (1.0, 1.4) 0.023 | 1.2 (1.0, 1.5) 0.048 |
| BODY.MASS.INDEX group recoded |  |  |  |
| 0 | 1.0 | 1.0 | 1.0 |
| 1 | 10.2 (2.0, 53.0) 0.005 | 9.9 (1.9, 52.6) 0.007 | 24.3 (3.0, 197.9) 0.003 |
| 2 | 5.8 (1.7, 19.3) 0.004 | 6.6 (1.9, 23.7) 0.004 | 12.5 (2.3, 68.4) 0.004 |

±íÖÐÊý¾Ý£º
¦Â (95%CI) Pvalue / OR (95%CI) Pvalue
½á¹û±äÁ¿: X1.MORT.OPERATIVE.MORTALITY.0.NONE.1YES
±©Â¶±äÁ¿: BODY.MASS.INDEX; BODY.MASS.INDEX group recoded
 Non-adjusted model adjust for: None
 Adjust I model adjust for: SMOKING.YES.0NO.1YES; SEX.0.FEMALE.1.MALE; AGE
 Adjust II model adjust for: PRIOR.SURGERY.0NO.1CABG.2VALVE.3OTHER; CEREBROVASCULAR.DISEASE.0NO.1YES; CHRONIC.RENAL.FAILURE.0NO.1YES; DIABETES.0NO.1YES; SMOKING.YES.0NO.1YES; SEX.0.FEMALE.1.MALE; AGE; RBC.U; PUMP.TIME; CROSS.CLAMP.TIME; PH; EF; OPERATION.TIME
¸÷Ä£ÐÍËùÓÃµÄÑù±¾Á¿

|  |  |  |  |  |
| --- | --- | --- | --- | --- |
| Outcome | Exposure | Non-adjusted | Adjust I | Adjust II |
| X1.MORT.OPERATIVE.MORTALITY.0.NONE.1YES | BODY.MASS.INDEX | 202 | 202 | 196 |
| X1.MORT.OPERATIVE.MORTALITY.0.NONE.1YES | BODY.MASS.INDEX group recoded | 202 | 202 | 196 |

´Ë±íÓÃÒ×õÍ³¼ÆÈí¼þ (www.empowerstats.com) ºÍRÈí¼þÉú³É£¬Éú³ÉÈÕÆÚ£º 2022-03-21
