# Supplementary material for: Body mass index and postoperative mortality in patients undergoing coronary artery bypass graft surgery plus valve replacement: a retrospective cohort study
Source: PeerJ. 2022 Jun 14;10:e13601. doi: 10.7717/peerj.13601 (PMC9205315; doi:10.7717/peerj.13601)
Supplement: Supplemental Information 4 [file peerj-10-13601-s004.zip › 3/1_3_tbl/1_3_tbl.htm]

## ÑÐ¾¿ÈËÈºÃèÊö

|  |  |  |  |  |  |
| --- | --- | --- | --- | --- | --- |
| BODY.MASS.INDEX group | <18 | >=18, <25 | >=25 | P-value | P-value\* |
| N | 12 | 127 | 63 |  |  |
| BODY.MASS.INDEX | 16.8 ± 0.9 | 22.1 ± 1.7 | 27.3 ± 1.7 | <0.001 | <0.001 |
| AGE | 65.9 ± 4.8 | 64.7 ± 8.7 | 60.8 ± 8.4 | 0.007 | 0.006 |
| RBC.U | 5.5 ± 3.8 | 5.2 ± 4.3 | 3.4 ± 2.9 | 0.009 | 0.008 |
| PUMP.TIME | 154.1 ± 47.3 | 156.2 ± 52.1 | 157.0 ± 40.1 | 0.981 | 0.958 |
| CROSS.CLAMP.TIME | 71.7 ± 23.2 | 69.2 ± 30.5 | 63.9 ± 30.6 | 0.471 | 0.204 |
| BNP | 3476.6 ± 3003.6 | 2156.6 ± 3202.1 | 1357.7 ± 1703.7 | 0.122 | 0.049 |
| BUN | 9.9 ± 5.7 | 19.7 ± 76.0 | 8.6 ± 3.6 | 0.486 | 0.811 |
| PH | 37.3 ± 9.9 | 41.1 ± 17.9 | 38.2 ± 16.9 | 0.466 | 0.227 |
| EF | 58.0 ± 11.4 | 61.7 ± 9.8 | 60.3 ± 9.7 | 0.356 | 0.380 |
| OPERATION.TIME | 8.1 ± 7.9 | 5.8 ± 1.7 | 6.4 ± 2.5 | 0.012 | 0.673 |
| PRIOR.SURGERY.0NO.1CABG.2VALVE.3OTHER |  |  |  | 0.737 | 0.717 |
| 0 | 10 (83.3%) | 101 (79.5%) | 49 (79.0%) |  |  |
| 2 | 0 (0.0%) | 1 (0.8%) | 2 (3.2%) |  |  |
| 3 | 2 (16.7%) | 25 (19.7%) | 11 (17.7%) |  |  |
| CEREBROVASCULAR.DISEASE.0NO.1YES |  |  |  | 0.577 | - |
| 0 | 11 (91.7%) | 105 (82.7%) | 50 (79.4%) |  |  |
| 1 | 1 (8.3%) | 22 (17.3%) | 13 (20.6%) |  |  |
| CHRONIC.RENAL.FAILURE.0NO.1YES |  |  |  | 0.788 | 0.884 |
| 0 | 12 (100.0%) | 116 (91.3%) | 59 (93.7%) |  |  |
| 1 | 0 (0.0%) | 10 (7.9%) | 4 (6.3%) |  |  |
| 2 | 0 (0.0%) | 1 (0.8%) | 0 (0.0%) |  |  |
| DIABETES.0NO.1YES |  |  |  | 0.144 | - |
| 0 | 12 (100.0%) | 112 (88.2%) | 51 (81.0%) |  |  |
| 1 | 0 (0.0%) | 15 (11.8%) | 12 (19.0%) |  |  |
| SMOKING.YES.0NO.1YES |  |  |  | 0.802 | - |
| 0 | 10 (83.3%) | 104 (81.9%) | 54 (85.7%) |  |  |
| 1 | 2 (16.7%) | 23 (18.1%) | 9 (14.3%) |  |  |
| SEX.0.FEMALE.1.MALE |  |  |  | 0.068 | - |
| 0 | 6 (50.0%) | 52 (40.9%) | 16 (25.4%) |  |  |
| 1 | 6 (50.0%) | 75 (59.1%) | 47 (74.6%) |  |  |
| X1.MORT.OPERATIVE.MORTALITY.0.NONE.1YES |  |  |  | 0.001 | - |
| 0 | 9 (75.0%) | 123 (96.9%) | 53 (84.1%) |  |  |
| 1 | 3 (25.0%) | 4 (3.1%) | 10 (15.9%) |  |  |
| AGE group |  |  |  | 0.025 | - |
| <60 | 1 (8.3%) | 31 (24.4%) | 25 (39.7%) |  |  |
| >=60 | 11 (91.7%) | 96 (75.6%) | 38 (60.3%) |  |  |
| EF group |  |  |  | 0.173 | - |
| <55 | 5 (41.7%) | 26 (20.6%) | 18 (28.6%) |  |  |
| >=55 | 7 (58.3%) | 100 (79.4%) | 45 (71.4%) |  |  |

±íÖÐ½á¹û:
Mean+SD / N(%)
PÖµ\*: ÈçÊÇÁ¬Ðø±äÁ¿£¬ÓÃKruskal WallisÖÈºÍ¼ìÑéµÃ³ö, Èç¼ÆÊý±äÁ¿ÓÐÀíÂÛÊý<10£¬ÓÃFisher¾«È·¸ÅÂÊ¼ìÑéµÃ³ö.
´Ë±íÓÃÒ×õÍ³¼ÆÈí¼þ (www.empowerstats.com) ºÍRÈí¼þÉú³É£¬Éú³ÉÈÕÆÚ£º 2022-03-21
