# Supplementary material for: Body mass index and postoperative mortality in patients undergoing coronary artery bypass graft surgery plus valve replacement: a retrospective cohort study
Source: PeerJ. 2022 Jun 14;10:e13601. doi: 10.7717/peerj.13601 (PMC9205315; doi:10.7717/peerj.13601)
Supplement: Supplemental Information 4 [file peerj-10-13601-s004.zip › 3/1_4_tbl/1_4_tbl.htm]

## ÑÐ¾¿ÈËÈºÃèÊö

|  |  |
| --- | --- |
|  |  |
|  | Mean+SD |
| BODY.MASS.INDEX | 23.4 ± 3.4 |
| AGE | 63.6 ± 8.6 |
| RBC.U | 4.6 ± 4.0 |
| PUMP.TIME | 156.3 ± 48.1 |
| CROSS.CLAMP.TIME | 67.7 ± 30.1 |
| BNP | 1995.5 ± 2821.0 |
| BUN | 15.6 ± 60.2 |
| PH | 40.0 ± 17.2 |
| EF | 61.0 ± 9.9 |
| OPERATION.TIME | 6.1 ± 2.7 |
|  | N (%) |
| PRIOR.SURGERY.0NO.1CABG.2VALVE.3OTHER |  |
| 0 | 160 (79.6%) |
| 2 | 3 (1.5%) |
| 3 | 38 (18.9%) |
| CEREBROVASCULAR.DISEASE.0NO.1YES |  |
| 0 | 166 (82.2%) |
| 1 | 36 (17.8%) |
| CHRONIC.RENAL.FAILURE.0NO.1YES |  |
| 0 | 187 (92.6%) |
| 1 | 14 (6.9%) |
| 2 | 1 (0.5%) |
| DIABETES.0NO.1YES |  |
| 0 | 175 (86.6%) |
| 1 | 27 (13.4%) |
| SMOKING.YES.0NO.1YES |  |
| 0 | 168 (83.2%) |
| 1 | 34 (16.8%) |
| SEX.0.FEMALE.1.MALE |  |
| 0 | 74 (36.6%) |
| 1 | 128 (63.4%) |
| X1.MORT.OPERATIVE.MORTALITY.0.NONE.1YES |  |
| 0 | 185 (91.6%) |
| 1 | 17 (8.4%) |
| AGE group |  |
| <60 | 57 (28.2%) |
| >=60 | 145 (71.8%) |
| EF group |  |
| <55 | 49 (24.4%) |
| >=55 | 152 (75.6%) |

±íÖÐ½á¹û:
Mean+SD / N(%)
´Ë±íÓÃÒ×õÍ³¼ÆÈí¼þ (www.empowerstats.com) ºÍRÈí¼þÉú³É£¬Éú³ÉÈÕÆÚ£º 2022-03-21
