# Supplementary material for: Body mass index and postoperative mortality in patients undergoing coronary artery bypass graft surgery plus valve replacement: a retrospective cohort study
Source: PeerJ. 2022 Jun 14;10:e13601. doi: 10.7717/peerj.13601 (PMC9205315; doi:10.7717/peerj.13601)
Supplement: Supplemental Information 4 [file peerj-10-13601-s004.zip › 3/1_5_tbl/1_5_tbl.htm]

## µ¥ÒòËØ·ÖÎö

|  |  |  |
| --- | --- | --- |
|  | Statistics | X1.MORT.OPERATIVE.MORTALITY.0.NONE.1YES |
| PRIOR.SURGERY.0NO.1CABG.2VALVE.3OTHER |  |  |
| 0 | 160 (79.6%) | 1.0 |
| 2 | 3 (1.5%) | 0.0 (0.0, Inf) 0.992 |
| 3 | 38 (18.9%) | 0.5 (0.1, 2.5) 0.423 |
| CEREBROVASCULAR.DISEASE.0NO.1YES |  |  |
| 0 | 166 (82.2%) | 1.0 |
| 1 | 36 (17.8%) | 2.8 (1.0, 8.2) 0.057 |
| CHRONIC.RENAL.FAILURE.0NO.1YES |  |  |
| 0 | 187 (92.6%) | 1.0 |
| 1 | 14 (6.9%) | 0.8 (0.1, 6.7) 0.855 |
| 2 | 1 (0.5%) | 0.0 (0.0, Inf) 0.993 |
| DIABETES.0NO.1YES |  |  |
| 0 | 175 (86.6%) | 1.0 |
| 1 | 27 (13.4%) | 3.1 (1.0, 9.6) 0.051 |
| SMOKING.YES.0NO.1YES |  |  |
| 0 | 168 (83.2%) | 1.0 |
| 1 | 34 (16.8%) | 0.3 (0.0, 2.2) 0.235 |
| BODY.MASS.INDEX | 23.4 ± 3.4 | 1.2 (1.0, 1.4) 0.028 |
| SEX.0.FEMALE.1.MALE |  |  |
| 0 | 74 (36.6%) | 1.0 |
| 1 | 128 (63.4%) | 0.6 (0.2, 1.7) 0.355 |
| AGE | 63.6 ± 8.6 | 1.0 (0.9, 1.1) 0.866 |
| RBC.U | 4.6 ± 4.0 | 1.0 (0.9, 1.2) 0.410 |
| PUMP.TIME | 156.3 ± 48.1 | 1.0 (1.0, 1.0) 0.057 |
| CROSS.CLAMP.TIME | 67.7 ± 30.1 | 1.0 (1.0, 1.0) 0.009 |
| BNP | 1995.5 ± 2821.0 | 1.0 (1.0, 1.0) 0.598 |
| BUN | 15.6 ± 60.2 | 1.0 (1.0, 1.0) 0.742 |
| PH | 40.0 ± 17.2 | 1.0 (1.0, 1.0) 0.994 |
| EF | 61.0 ± 9.9 | 1.0 (0.9, 1.0) 0.207 |
| OPERATION.TIME | 6.1 ± 2.7 | 1.0 (0.9, 1.2) 0.542 |
| BODY.MASS.INDEX group |  |  |
| <18 | 12 (5.9%) | 1.0 |
| >=18, <25 | 127 (62.9%) | 0.1 (0.0, 0.5) 0.005 |
| >=25 | 63 (31.2%) | 0.6 (0.1, 2.5) 0.448 |
| AGE group |  |  |
| <60 | 57 (28.2%) | 1.0 |
| >=60 | 145 (71.8%) | 0.9 (0.3, 2.8) 0.909 |
| EF group |  |  |
| <55 | 49 (24.4%) | 1.0 |
| >=55 | 152 (75.6%) | 0.4 (0.2, 1.2) 0.100 |

±íÖÐÊý¾Ý£º
½á¹û±äÁ¿: X1.MORT.OPERATIVE.MORTALITY.0.NONE.1YES
±©Â¶±äÁ¿: PRIOR.SURGERY.0NO.1CABG.2VALVE.3OTHER; CEREBROVASCULAR.DISEASE.0NO.1YES; CHRONIC.RENAL.FAILURE.0NO.1YES; DIABETES.0NO.1YES; SMOKING.YES.0NO.1YES; BODY.MASS.INDEX; SEX.0.FEMALE.1.MALE; AGE; RBC.U; PUMP.TIME; CROSS.CLAMP.TIME; BNP; BUN; PH; EF; OPERATION.TIME; BODY.MASS.INDEX group; AGE group; EF group
µ÷Õû±äÁ¿: None
¸÷Ä£ÐÍËùÓÃµÄÑù±¾Á¿

|  |  |
| --- | --- |
| Exposure | X1.MORT.OPERATIVE.MORTALITY.0.NONE.1YES |
| PRIOR.SURGERY.0NO.1CABG.2VALVE.3OTHER | 201 |
| CEREBROVASCULAR.DISEASE.0NO.1YES | 202 |
| CHRONIC.RENAL.FAILURE.0NO.1YES | 202 |
| DIABETES.0NO.1YES | 202 |
| SMOKING.YES.0NO.1YES | 202 |
| BODY.MASS.INDEX | 202 |
| SEX.0.FEMALE.1.MALE | 202 |
| AGE | 202 |
| RBC.U | 202 |
| PUMP.TIME | 198 |
| CROSS.CLAMP.TIME | 198 |
| BNP | 108 |
| BUN | 189 |
| PH | 201 |
| EF | 201 |
| OPERATION.TIME | 202 |
| BODY.MASS.INDEX group | 202 |
| AGE group | 202 |
| EF group | 201 |

´Ë±íÓÃÒ×õÍ³¼ÆÈí¼þ (www.empowerstats.com) ºÍRÈí¼þÉú³É£¬Éú³ÉÈÕÆÚ£º 2022-03-21
