# Supplementary material for: Body mass index and postoperative mortality in patients undergoing coronary artery bypass graft surgery plus valve replacement: a retrospective cohort study
Source: PeerJ. 2022 Jun 14;10:e13601. doi: 10.7717/peerj.13601 (PMC9205315; doi:10.7717/peerj.13601)
Supplement: Supplemental Information 4 [file peerj-10-13601-s004.zip › 3/1_6_tbl/1_6_tbl.htm]

## ·Ö²ã·ÖÎö

Stratified variables should be categorical

|  |  |  |
| --- | --- | --- |
| X= BODY.MASS.INDEX |  |  |
|  | N | X1.MORT.OPERATIVE.MORTALITY.0.NONE.1YES |
| BODY.MASS.INDEX group |  |  |
| <18 | 12 | 0.5 (0.1, 2.5) 0.437 |
| >=18, <25 | 127 | 0.8 (0.4, 1.4) 0.351 |
| >=25 | 63 | 2.6 (1.5, 4.6) 0.001 |
| PRIOR.SURGERY.0NO.1CABG.2VALVE.3OTHER |  |  |
| 0 | 160 | 1.2 (1.0, 1.4) 0.030 |
| 2 | 3 |  |
| 3 | 38 | 1.1 (0.7, 1.8) 0.559 |
| CEREBROVASCULAR.DISEASE.0NO.1YES |  |  |
| 0 | 166 | 1.2 (1.0, 1.4) 0.080 |
| 1 | 36 | 1.2 (0.9, 1.5) 0.322 |
| CHRONIC.RENAL.FAILURE.0NO.1YES |  |  |
| 0 | 187 | 1.2 (1.0, 1.4) 0.054 |
| 1 | 14 | 1.6 (0.6, 4.3) 0.331 |
| 2 | 1 |  |
| DIABETES.0NO.1YES |  |  |
| 0 | 175 | 1.1 (0.9, 1.3) 0.305 |
| 1 | 27 | 1.4 (1.0, 2.0) 0.058 |
| SMOKING.YES.0NO.1YES |  |  |
| 0 | 168 | 1.2 (1.0, 1.4) 0.042 |
| 1 | 34 | 1.4 (0.7, 2.9) 0.352 |
| SEX.0.FEMALE.1.MALE |  |  |
| 0 | 74 | 1.1 (0.9, 1.4) 0.205 |
| 1 | 128 | 1.3 (1.0, 1.6) 0.046 |
| AGE group |  |  |
| 18 - 60 | 60 | 1.4 (1.0, 2.0) 0.048 |
| 61 - 67 | 72 | 1.3 (1.0, 1.7) 0.070 |
| 68 - 82 | 70 | 1.0 (0.8, 1.3) 0.951 |
| RBC.U group |  |  |
| 0 - 2.5 | 58 | 40.7 (0.3, 6047.8) 0.146 |
| 3 - 4.5 | 74 | 1.1 (0.9, 1.3) 0.506 |
| 5 - 22 | 70 | 1.1 (0.9, 1.4) 0.266 |
| PUMP.TIME group |  |  |
| 2.3 - 135 | 65 | 0.9 (0.6, 1.4) 0.709 |
| 136 - 174 | 67 | 1.3 (1.0, 1.6) 0.045 |
| 175 - 360 | 66 | 1.1 (0.9, 1.5) 0.289 |
| CROSS.CLAMP.TIME group |  |  |
| 27 - 50 | 62 | 0.9 (0.7, 1.4) 0.737 |
| 51 - 73 | 68 | 1.6 (1.1, 2.3) 0.014 |
| 74 - 210 | 68 | 1.1 (0.9, 1.4) 0.294 |
| BNP group |  |  |
| 18.19 - 647 | 36 | 2.1 (1.0, 4.4) 0.056 |
| 651 - 1600 | 36 | 0.9 (0.6, 1.3) 0.462 |
| 1608 - 21893 | 36 | 0.9 (0.6, 1.3) 0.500 |
| BUN group |  |  |
| 3 - 5.65 | 35 | 1.1 (0.8, 1.5) 0.657 |
| 6 - 9.49 | 88 | 1.4 (1.0, 1.7) 0.021 |
| 10 - 709 | 66 | 1.0 (0.8, 1.3) 0.828 |
| PH group |  |  |
| 1 - 31 | 62 | 1.3 (0.9, 1.8) 0.173 |
| 32 - 41 | 69 | 1.1 (0.9, 1.4) 0.404 |
| 42 - 110 | 70 | 1.2 (1.0, 1.6) 0.083 |
| EF group |  |  |
| 31 - 58 | 64 | 1.4 (1.1, 1.7) 0.008 |
| 59 - 65 | 59 | 0.5 (0.2, 1.0) 0.061 |
| 66 - 78 | 78 | 1.1 (0.9, 1.5) 0.373 |
| OPERATION.TIME group |  |  |
| 2.3 - 5.1 | 63 | 1.1 (0.9, 1.4) 0.275 |
| 5.2 - 6.7 | 71 | 1.3 (0.9, 1.8) 0.206 |
| 6.75 - 33 | 68 | 1.3 (1.0, 1.7) 0.104 |
| AGE group |  |  |
| <60 | 57 | 1.4 (1.0, 1.9) 0.055 |
| >=60 | 145 | 1.1 (0.9, 1.4) 0.180 |
| EF group |  |  |
| <55 | 49 | 1.2 (1.0, 1.6) 0.072 |
| >=55 | 152 | 1.1 (0.9, 1.4) 0.206 |

±íÖÐÊý¾Ý£º
¦Â (95%CI) Pvalue / OR (95%CI) Pvalue
½á¹û±äÁ¿: X1.MORT.OPERATIVE.MORTALITY.0.NONE.1YES
±©Â¶±äÁ¿: BODY.MASS.INDEX
µ÷Õû±äÁ¿: None
¸÷Ä£ÐÍËùÓÃµÄÑù±¾Á¿

|  |  |  |
| --- | --- | --- |
| Exposure | Sub-group | X1.MORT.OPERATIVE.MORTALITY.0.NONE.1YES |
| BODY.MASS.INDEX | BODY.MASS.INDEX group = <18 | 12 |
| BODY.MASS.INDEX | BODY.MASS.INDEX group = >=18, <25 | 127 |
| BODY.MASS.INDEX | BODY.MASS.INDEX group = >=25 | 63 |
| BODY.MASS.INDEX | PRIOR.SURGERY.0NO.1CABG.2VALVE.3OTHER = 0 | 160 |
| BODY.MASS.INDEX | PRIOR.SURGERY.0NO.1CABG.2VALVE.3OTHER = 2 | 3 |
| BODY.MASS.INDEX | PRIOR.SURGERY.0NO.1CABG.2VALVE.3OTHER = 3 | 38 |
| BODY.MASS.INDEX | CEREBROVASCULAR.DISEASE.0NO.1YES = 0 | 166 |
| BODY.MASS.INDEX | CEREBROVASCULAR.DISEASE.0NO.1YES = 1 | 36 |
| BODY.MASS.INDEX | CHRONIC.RENAL.FAILURE.0NO.1YES = 0 | 187 |
| BODY.MASS.INDEX | CHRONIC.RENAL.FAILURE.0NO.1YES = 1 | 14 |
| BODY.MASS.INDEX | CHRONIC.RENAL.FAILURE.0NO.1YES = 2 | 1 |
| BODY.MASS.INDEX | DIABETES.0NO.1YES = 0 | 175 |
| BODY.MASS.INDEX | DIABETES.0NO.1YES = 1 | 27 |
| BODY.MASS.INDEX | SMOKING.YES.0NO.1YES = 0 | 168 |
| BODY.MASS.INDEX | SMOKING.YES.0NO.1YES = 1 | 34 |
| BODY.MASS.INDEX | SEX.0.FEMALE.1.MALE = 0 | 74 |
| BODY.MASS.INDEX | SEX.0.FEMALE.1.MALE = 1 | 128 |
| BODY.MASS.INDEX | AGE group = 18 - 60 | 60 |
| BODY.MASS.INDEX | AGE group = 61 - 67 | 72 |
| BODY.MASS.INDEX | AGE group = 68 - 82 | 70 |
| BODY.MASS.INDEX | RBC.U group = 0 - 2.5 | 58 |
| BODY.MASS.INDEX | RBC.U group = 3 - 4.5 | 74 |
| BODY.MASS.INDEX | RBC.U group = 5 - 22 | 70 |
| BODY.MASS.INDEX | PUMP.TIME group = 2.3 - 135 | 65 |
| BODY.MASS.INDEX | PUMP.TIME group = 136 - 174 | 67 |
| BODY.MASS.INDEX | PUMP.TIME group = 175 - 360 | 66 |
| BODY.MASS.INDEX | CROSS.CLAMP.TIME group = 27 - 50 | 62 |
| BODY.MASS.INDEX | CROSS.CLAMP.TIME group = 51 - 73 | 68 |
| BODY.MASS.INDEX | CROSS.CLAMP.TIME group = 74 - 210 | 68 |
| BODY.MASS.INDEX | BNP group = 18.19 - 647 | 36 |
| BODY.MASS.INDEX | BNP group = 651 - 1600 | 36 |
| BODY.MASS.INDEX | BNP group = 1608 - 21893 | 36 |
| BODY.MASS.INDEX | BUN group = 3 - 5.65 | 35 |
| BODY.MASS.INDEX | BUN group = 6 - 9.49 | 88 |
| BODY.MASS.INDEX | BUN group = 10 - 709 | 66 |
| BODY.MASS.INDEX | PH group = 1 - 31 | 62 |
| BODY.MASS.INDEX | PH group = 32 - 41 | 69 |
| BODY.MASS.INDEX | PH group = 42 - 110 | 70 |
| BODY.MASS.INDEX | EF group = 31 - 58 | 64 |
| BODY.MASS.INDEX | EF group = 59 - 65 | 59 |
| BODY.MASS.INDEX | EF group = 66 - 78 | 78 |
| BODY.MASS.INDEX | OPERATION.TIME group = 2.3 - 5.1 | 63 |
| BODY.MASS.INDEX | OPERATION.TIME group = 5.2 - 6.7 | 71 |
| BODY.MASS.INDEX | OPERATION.TIME group = 6.75 - 33 | 68 |
| BODY.MASS.INDEX | AGE group = <60 | 57 |
| BODY.MASS.INDEX | AGE group = >=60 | 145 |
| BODY.MASS.INDEX | EF group = <55 | 49 |
| BODY.MASS.INDEX | EF group = >=55 | 152 |

´Ë±íÓÃÒ×õÍ³¼ÆÈí¼þ(www.empowerstats.com) ºÍRÈí¼þÉú³É£¬Éú³ÉÈÕÆÚ£º 2022-03-21
