# Supplementary material for: Body mass index and postoperative mortality in patients undergoing coronary artery bypass graft surgery plus valve replacement: a retrospective cohort study
Source: PeerJ. 2022 Jun 14;10:e13601. doi: 10.7717/peerj.13601 (PMC9205315; doi:10.7717/peerj.13601)
Supplement: Supplemental Information 4 [file peerj-10-13601-s004.zip › 3/1_7_tbl/1_7_tbl.htm]

## Ð­±äÁ¿¼ì²éÓëÉ¸Ñ¡

VIF ¹²ÏßÐÔÉ¸²é

|  |  |
| --- | --- |
|  | Step 1 |
| BODY.MASS.INDEX | 1.4 |
| PRIOR.SURGERY.0NO.1CABG.2VALVE.3OTHER | 1.3 |
| CEREBROVASCULAR.DISEASE.0NO.1YES | 1.3 |
| CHRONIC.RENAL.FAILURE.0NO.1YES | 1.5 |
| DIABETES.0NO.1YES | 1.2 |
| SMOKING.YES.0NO.1YES | 1.3 |
| SEX.0.FEMALE.1.MALE | 1.2 |
| AGE | 1.4 |
| RBC.U | 1.5 |
| PUMP.TIME | 1.3 |
| CROSS.CLAMP.TIME | 1.2 |
| BNP | 1.6 |
| BUN | 1.2 |
| PH | 1.2 |
| EF | 1.2 |
| OPERATION.TIME | 1.3 |

Y= X1.MORT.OPERATIVE.MORTALITY.0.NONE.1YES
1. Öð¸ö²é¿´Ð­±äÁ¿ÓëYµÄ¹ØÏµ

|  |  |  |  |  |  |  |  |  |
| --- | --- | --- | --- | --- | --- | --- | --- | --- |
| Covariates | N | term | beta | Se. | exp(beta) | 95%CI Low | 95%CI Upp | P.value |
| PRIOR.SURGERY.0NO.1CABG.2VALVE.3OTHER | 201 | factor(PRIOR.SURGERY.0NO.1CABG.2VALVE.3OTHER)2 | -14.2974 | 1385.3778 | 0.0000 | 0.0000 | Inf | 0.9918 |
|  |  | factor(PRIOR.SURGERY.0NO.1CABG.2VALVE.3OTHER)3 | -0.6217 | 0.7755 | 0.5370 | 0.1175 | 2.4552 | 0.4227 |
| CEREBROVASCULAR.DISEASE.0NO.1YES | 202 | factor(CEREBROVASCULAR.DISEASE.0NO.1YES)1 | 1.0361 | 0.5453 | 2.8182 | 0.9678 | 8.2062 | 0.0574 |
| CHRONIC.RENAL.FAILURE.0NO.1YES | 202 | factor(CHRONIC.RENAL.FAILURE.0NO.1YES)1 | -0.1959 | 1.0702 | 0.8221 | 0.1009 | 6.6970 | 0.8548 |
|  |  | factor(CHRONIC.RENAL.FAILURE.0NO.1YES)2 | -13.1970 | 1455.3976 | 0.0000 | 0.0000 | Inf | 0.9928 |
| DIABETES.0NO.1YES | 202 | factor(DIABETES.0NO.1YES)1 | 1.1272 | 0.5787 | 3.0871 | 0.9930 | 9.5979 | 0.0514 |
| SMOKING.YES.0NO.1YES | 202 | factor(SMOKING.YES.0NO.1YES)1 | -1.2452 | 1.0485 | 0.2879 | 0.0369 | 2.2476 | 0.2350 |
| SEX.0.FEMALE.1.MALE | 202 | factor(SEX.0.FEMALE.1.MALE)1 | -0.4717 | 0.5096 | 0.6239 | 0.2298 | 1.6940 | 0.3546 |
| AGE | 202 | AGE | -0.0049 | 0.0292 | 0.9951 | 0.9398 | 1.0537 | 0.8663 |
| RBC.U | 202 | RBC.U | 0.0476 | 0.0578 | 1.0487 | 0.9364 | 1.1746 | 0.4105 |
| PUMP.TIME | 198 | PUMP.TIME | 0.0094 | 0.0049 | 1.0094 | 0.9997 | 1.0192 | 0.0571 |
| CROSS.CLAMP.TIME | 198 | CROSS.CLAMP.TIME | 0.0177 | 0.0068 | 1.0179 | 1.0045 | 1.0314 | 0.0087 |
| BNP | 108 | BNP | -0.0001 | 0.0002 | 0.9999 | 0.9995 | 1.0003 | 0.5975 |
| BUN | 189 | BUN | -0.0044 | 0.0134 | 0.9956 | 0.9698 | 1.0221 | 0.7424 |
| PH | 201 | PH | 0.0001 | 0.0148 | 1.0001 | 0.9716 | 1.0295 | 0.9940 |
| EF | 201 | EF | -0.0302 | 0.0239 | 0.9703 | 0.9258 | 1.0168 | 0.2069 |
| OPERATION.TIME | 202 | OPERATION.TIME | 0.0440 | 0.0721 | 1.0450 | 0.9072 | 1.2036 | 0.5419 |

2. ÔÚ»ù±¾Ä£ÐÍÖÐÒý½øÐ­±äÁ¿ÓëÔÚÍêÕûÄ£ÐÍÖÐÌÞ³ýÐ­±äÁ¿, ¹Û²ìXµÄ»Ø¹éÏµÊýµÄ±ä»¯
X= BODY.MASS.INDEX

|  |  |  |  |  |
| --- | --- | --- | --- | --- |
|  |  | »ù±¾Ä£ÐÍ | ÍêÕûÄ£ÐÍ |  |
| Ð­±äÁ¿ | +/- term | BODY.MASS.INDEX | BODY.MASS.INDEX | Ñ¡³ö |
|  | ÆðÊ¼»Ø¹éÏµÊý | 0.0605 | -0.1075 |  |
| PRIOR.SURGERY.0NO.1CABG.2VALVE.3OTHER | factor(PRIOR.SURGERY.0NO.1CABG.2VALVE.3OTHER) | 0.0652 | -0.1228 \* | Yes |
| CEREBROVASCULAR.DISEASE.0NO.1YES | factor(CEREBROVASCULAR.DISEASE.0NO.1YES) | 0.0570 | 0.0356 \* | Yes |
| CHRONIC.RENAL.FAILURE.0NO.1YES | factor(CHRONIC.RENAL.FAILURE.0NO.1YES) | 0.0565 | 0.0663 \* | Yes |
| DIABETES.0NO.1YES | factor(DIABETES.0NO.1YES) | 0.0231 \* | 0.0459 \* | Yes |
| SMOKING.YES.0NO.1YES | factor(SMOKING.YES.0NO.1YES) | 0.0568 | -0.0507 \* | Yes |
| SEX.0.FEMALE.1.MALE | factor(SEX.0.FEMALE.1.MALE) | 0.0748 \* | -0.0874 \* | Yes |
| AGE | AGE | 0.0840 \* | -0.0847 \* | Yes |
| RBC.U | RBC.U | 0.1060 \* | -0.0720 \* | Yes |
| PUMP.TIME | PUMP.TIME | 0.0843 \* | -0.1245 \* | Yes |
| CROSS.CLAMP.TIME | CROSS.CLAMP.TIME | 0.0528 \* | 0.0604 \* | Yes |
| BNP | BNP | 0.0545 | 0.0566 \* | Yes |
| BUN | BUN | 0.0604 | -0.1257 \* | Yes |
| PH | PH | 0.0725 \* | -0.0006 \* | Yes |
| EF | EF | 0.0515 \* | 0.0609 \* | Yes |
| OPERATION.TIME | OPERATION.TIME | 0.0663 | 0.0739 \* | Yes |

\* ±íÊ¾ÓëÆðÊ¼»Ø¹éÏµÊýÏà±È±ä»¯³¬¹ý 10%
É¸Ñ¡³öÀ´µÄÐ­±äÁ¿

|  |  |  |  |
| --- | --- | --- | --- |
| Y | X | Ñ¡³öµÄÐ­±äÁ¿£¨±ê×¼1£© | Ñ¡³öµÄÐ­±äÁ¿£¨±ê×¼2£© |
| X1.MORT.OPERATIVE.MORTALITY.0.NONE.1YES | BODY.MASS.INDEX | PRIOR.SURGERY.0NO.1CABG.2VALVE.3OTHER CEREBROVASCULAR.DISEASE.0NO.1YES CHRONIC.RENAL.FAILURE.0NO.1YES DIABETES.0NO.1YES SMOKING.YES.0NO.1YES SEX.0.FEMALE.1.MALE AGE RBC.U PUMP.TIME CROSS.CLAMP.TIME BNP BUN PH EF OPERATION.TIME | PRIOR.SURGERY.0NO.1CABG.2VALVE.3OTHER CEREBROVASCULAR.DISEASE.0NO.1YES CHRONIC.RENAL.FAILURE.0NO.1YES DIABETES.0NO.1YES SMOKING.YES.0NO.1YES SEX.0.FEMALE.1.MALE AGE RBC.U PUMP.TIME CROSS.CLAMP.TIME BNP BUN PH EF OPERATION.TIME |

×¢ÊÍ£º
1. ±ê×¼1£ºÔÚ»ù±¾Ä£ÐÍÖÐÒý½øÐ­±äÁ¿»òÔÚÍêÕûÄ£ÐÍÖÐÌÞ³ýÐ­±äÁ¿¶ÔXµÄ»Ø¹éÏµÊýµÄÓ°Ïì>10%
2. ±ê×¼2£º±ê×¼1»òÐ­±äÁ¿¶ÔYµÄ»Ø¹éÏµÊýPÖµ<0.1
Created by EmpowerStats (www.empowerstats.com) and R on 2022-03-21
