# Supplementary material for: Body mass index and postoperative mortality in patients undergoing coronary artery bypass graft surgery plus valve replacement: a retrospective cohort study
Source: PeerJ. 2022 Jun 14;10:e13601. doi: 10.7717/peerj.13601 (PMC9205315; doi:10.7717/peerj.13601)
Supplement: Supplemental Information 4 [file peerj-10-13601-s004.zip › 3/1_8_tbl/1_8_tbl.htm]

## ãÐÖµÐ§Ó¦·ÖÎö

For exposure: BODY.MASS.INDEX

|  |  |
| --- | --- |
| Outcome: | X1.MORT.OPERATIVE.MORTALITY.0.NONE.1YES |
| Ä£ÐÍ I |  |
| Ò»ÌõÖ±ÏßÐ§Ó¦ | 1.2 (1.0, 1.5) 0.048 |
| Ä£ÐÍ II |  |
| ÕÛµã(K1,K2) | 18, 25 |
| < K1 ¶ÎÐ§Ó¦ 1 | 0.2 (0.0, 1.1) 0.062 |
| K1-K2 ¶ÎÐ§Ó¦ 2 | 0.7 (0.4, 1.3) 0.277 |
| > K2 ¶ÎÐ§Ó¦ 3 | 4.2 (1.6, 11.5) 0.005 |
| 1Óë2µÄÐ§Ó¦²î | 0.3 (0.0, 2.4) 0.266 |
| 3Óë2µÄÐ§Ó¦²î | 6.0 (1.8, 19.9) 0.003 |
| ¶ÔÊýËÆÈ»±È¼ìÑé | <0.001 |

±íÖÐÊý¾Ý£º
¦Â (95%CI) Pvalue / OR (95%CI) Pvalue
½á¹û±äÁ¿: X1.MORT.OPERATIVE.MORTALITY.0.NONE.1YES
±©Â¶±äÁ¿: BODY.MASS.INDEX
µ÷Õû±äÁ¿: PRIOR.SURGERY.0NO.1CABG.2VALVE.3OTHER; CEREBROVASCULAR.DISEASE.0NO.1YES; CHRONIC.RENAL.FAILURE.0NO.1YES; DIABETES.0NO.1YES; SMOKING.YES.0NO.1YES; SEX.0.FEMALE.1.MALE; AGE; RBC.U; PUMP.TIME; CROSS.CLAMP.TIME; PH; EF; OPERATION.TIME
¸÷Ä£ÐÍËùÓÃµÄÑù±¾Á¿

|  |  |  |
| --- | --- | --- |
| Outcome | Exposure | N |
| X1.MORT.OPERATIVE.MORTALITY.0.NONE.1YES | BODY.MASS.INDEX | 196 |

´Ë±íÓÃÒ×õÍ³¼ÆÈí¼þ (www.empowerstats.com) ºÍRÈí¼þÉú³É£¬Éú³ÉÈÕÆÚ£º 2022-03-21
