# Supplementary figures and images for: Body mass index and postoperative mortality in patients undergoing coronary artery bypass graft surgery plus valve replacement: a retrospective cohort study
Source: PeerJ. 2022 Jun 14;10:e13601. doi: 10.7717/peerj.13601 (PMC9205315; doi:10.7717/peerj.13601)

X1.MORT.OPERATIVE.MORTALITY.0.NONE.1YES

1.2  
1.0  
0.8  
0.6  
0.4  
0.2  
0.0

15

20

25

30

BODY.MASS. INDEX

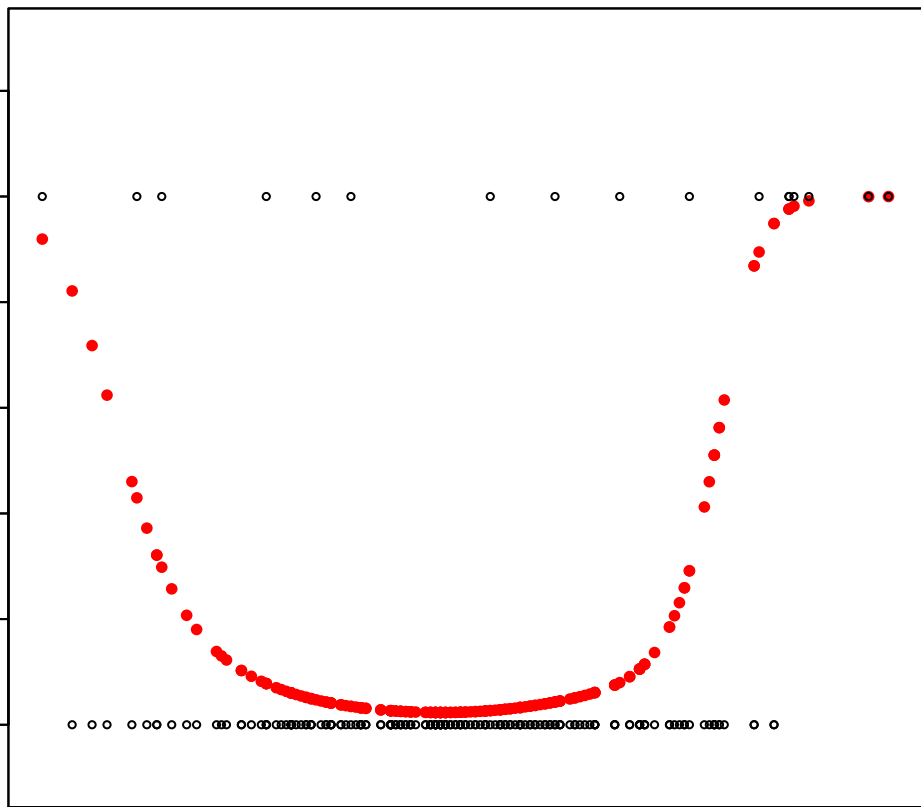

Supplement: Supplemental Information 4 [file peerj-10-13601-s004.zip › 3/1_1_tbl/1_1_tbl_X1.MORT.OPERATIVE.MORTALITY.0.NONE.1YES_BODY.MASS.INDEX_scatter.pdf]

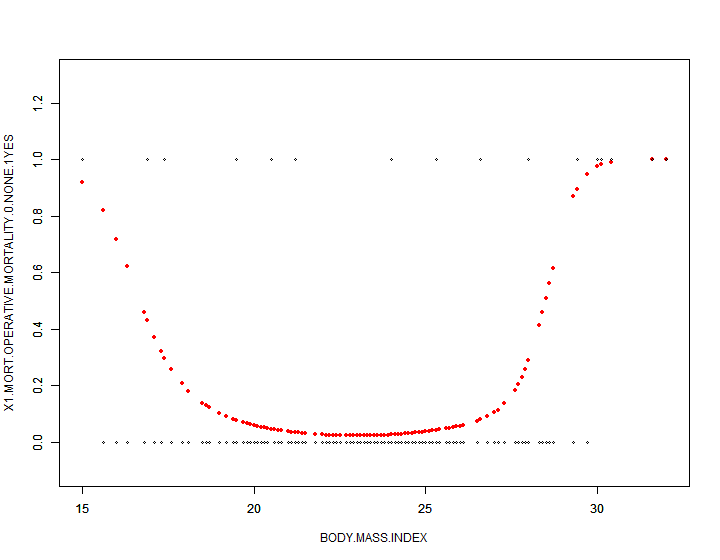

Supplement: Supplemental Information 4 [file peerj-10-13601-s004.zip › 3/1_1_tbl/1_1_tbl_X1.MORT.OPERATIVE.MORTALITY.0.NONE.1YES_BODY.MASS.INDEX_scatter.png]

X1.MORT.OPERATIVE.MORTALITY.0.NONE.1YES

1.0  
0.8  
0.6  
0.4  
0.2  
0.0

15

20

25

30

BODY.MASS. INDEX

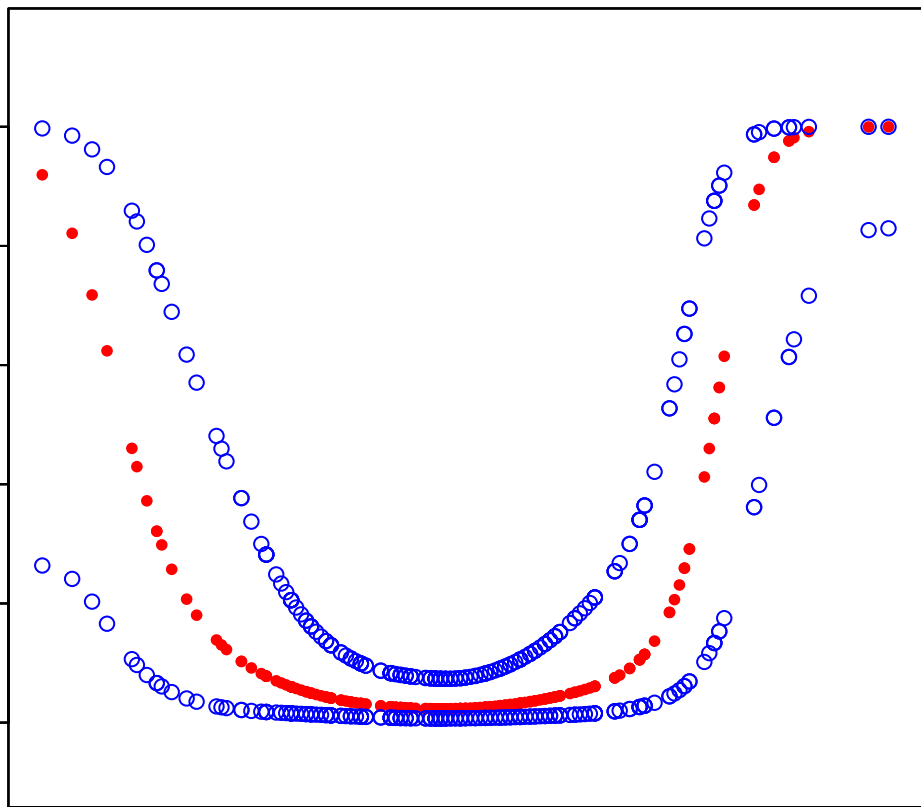

Supplement: Supplemental Information 4 [file peerj-10-13601-s004.zip › 3/1_1_tbl/1_1_tbl_X1.MORT.OPERATIVE.MORTALITY.0.NONE.1YES_BODY.MASS.INDEX_smooth.pdf]

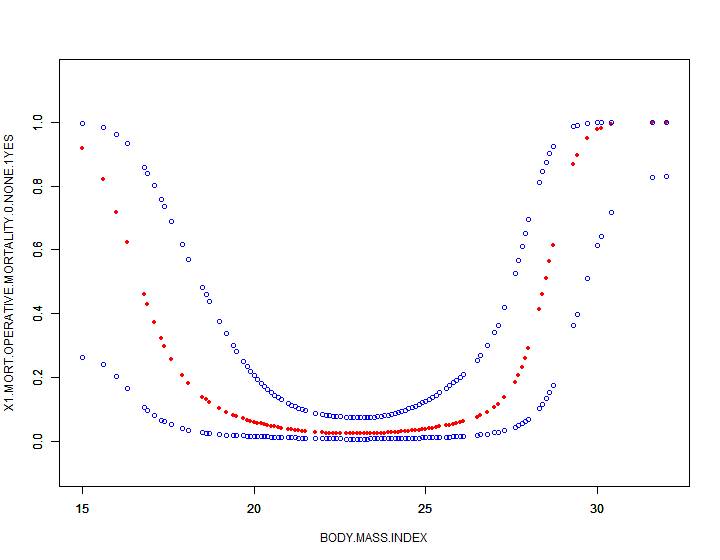

Supplement: Supplemental Information 4 [file peerj-10-13601-s004.zip › 3/1_1_tbl/1_1_tbl_X1.MORT.OPERATIVE.MORTALITY.0.NONE.1YES_BODY.MASS.INDEX_smooth.png]

X1.MORT.OPERATIVE.MORTALITY.0.NONE.1YES

1.0  
0.8  
0.6  
0.4  
0.2  
0.0

15

20

25

30

BODY.MASS. INDEX

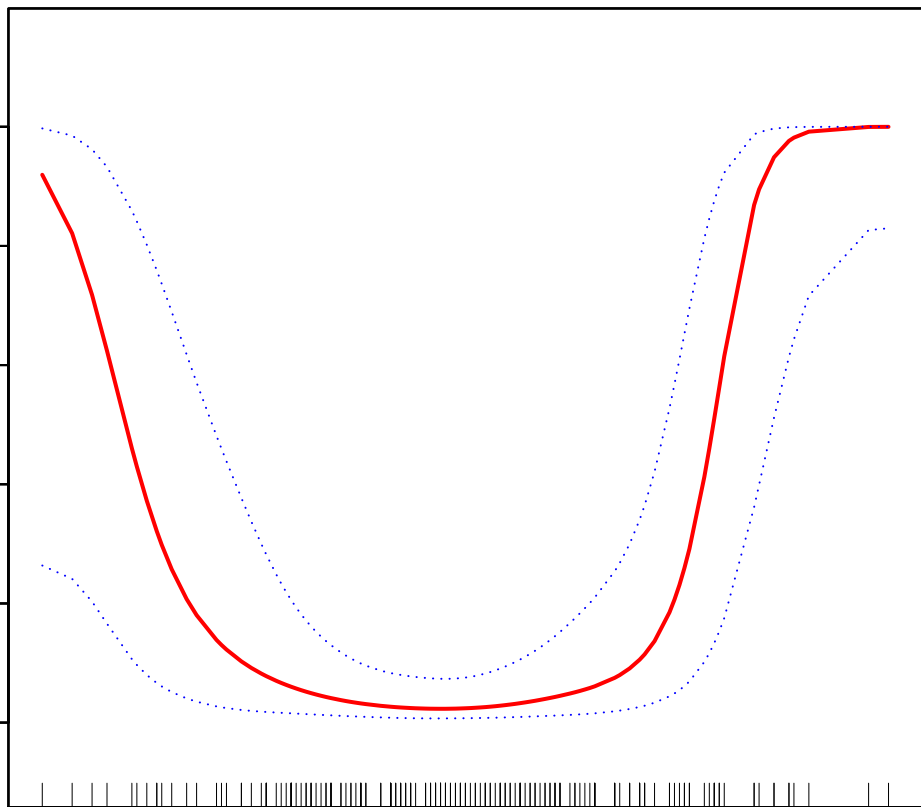

Supplement: Supplemental Information 4 [file peerj-10-13601-s004.zip › 3/1_1_tbl/1_1_tbl_X1.MORT.OPERATIVE.MORTALITY.0.NONE.1YES_BODY.MASS.INDEX_smooth1.pdf]

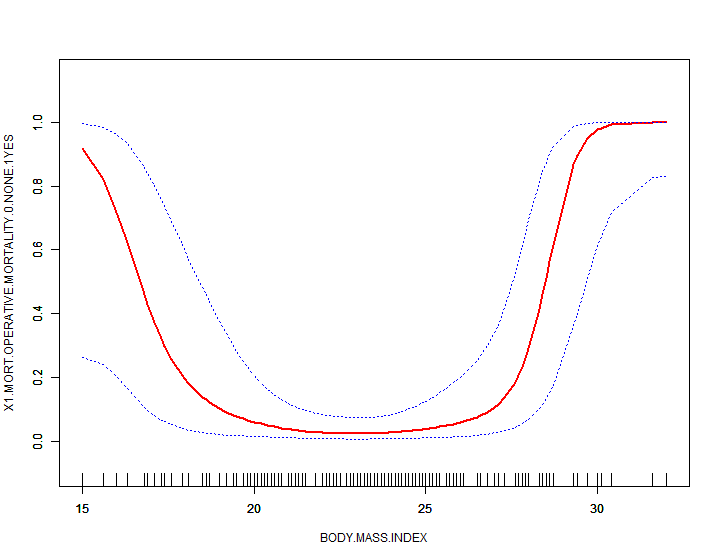

Supplement: Supplemental Information 4 [file peerj-10-13601-s004.zip › 3/1_1_tbl/1_1_tbl_X1.MORT.OPERATIVE.MORTALITY.0.NONE.1YES_BODY.MASS.INDEX_smooth1.png]

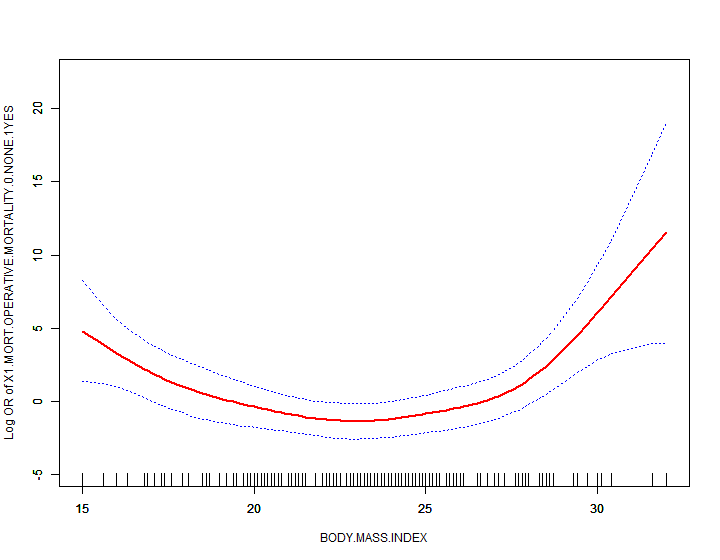

Supplement: Supplemental Information 4 [file peerj-10-13601-s004.zip › 3/1_1_tbl/1_1_tbl_X1.MORT.OPERATIVE.MORTALITY.0.NONE.1YES_BODY.MASS.INDEX_smooth2.png]
